# Gag P2/NC and pol genetic diversity, polymorphism, and drug resistance mutations in HIV-1 CRF02_AG- and non-CRF02_AG-infected patients in Yaoundé, Cameroon

**DOI:** 10.1038/s41598-017-14095-4

**Published:** 2017-10-26

**Authors:** Georges Teto, Claude T. Tagny, Dora Mbanya, Julius Y. Fonsah, Joseph Fokam, Emilienne Nchindap, Léopoldine Kenmogne, Alfred K. Njamnshi, Georgette D. Kanmogne

**Affiliations:** 1 Department of Pharmacology and Experimental Neuroscience, College of Medicine, University of Nebraska Medical Center, Omaha, NE USA; 2 Faculty of Medicine and Biomedical Sciences, The University of Yaoundé I, Yaoundé, Cameroon; 3 Yaoundé University Teaching Hospital, Yaoundé, Cameroon; 4 Department of Neurology, Yaoundé Central Hospital, Yaoundé, Cameroon; 5 Virology Laboratory, Chantal BIYA International Reference Centre for research on HIV/AIDS prevention and management, Yaoundé, Cameroon

## Abstract

In HIV-1 subtype-B, specific mutations in Gag cleavage sites (CS) are associated with treatment failure, with limited knowledge among non-B subtypes. We analyzed non-B HIV-1 gag and pol (protease/reverse-transcriptase) sequences from Cameroonians for drug resistance mutations (DRMs) in the gag P2/NC CS, and pol major DRMs. Phylogeny of the 141 sequences revealed a high genetic diversity (12 subtypes): 67.37% CRF02_AG versus 32.6% non-CRF02_AG. Overall, 7.3% transmitted and 34.3% acquired DRMs were found, including M184V, thymidine analogue mutations (T215F, D67N, K70R, K219Q), NNRTIs (L100I, Y181C, K103N, V108I, Y188L), and PIs (V82L). Twelve subjects [10 with HIV-1 CRF02_AG, 8 treatment-naïve and 4 on 3TC-AZT-NVP] showed 3 to 4 mutations in the Gag P2/NC CS: S373Q/T/A, A374T/S/G/N, T375S/A/N/G, I376V, G381S, and R380K. Subjects with or without Gag P2/NC CS mutations showed no significant difference in viral loads. Treatment-naïve subjects harboring NRTI-DRMs had significantly lower CD4 cells than those with NRTI-DRMs on ART (p = 0.042). Interestingly, two subjects had major DRMs to NRTIs, NNRTIs, and 4 mutations in the Gag P2/NC CS. In this prevailing CRF02_AG population with little exposure to PIs (~3%), mutations in the Gag P2/NC CS could increase the risk of treatment failure if there is increased use of PIs-based therapy.

## Introduction

Of the 37 million individuals worldwide currently living with HIV/AIDS, 70% are in Sub-Saharan Africa (SSA)^1^. With the high number of HIV/AIDS related deaths in SSA over the past three decades, there have been global efforts to increase access to antiretroviral therapy (ART)^2^. However, up to 75% of adults on ART do not achieve viral suppression in SSA^3,4^. The reasons for this non-viral suppression are multifactorial and included non-adherence to ART^3,5–8^, treatment interruptions^5,9,10^, and sustained high viremia^8,10,11^. These factors lead to the emergence of drug resistant HIV and risks of onward transmission of drug resistance mutations (DRMs)^12,13^.

With the current World Health Organization (WHO) guidelines that recommend treating all HIV-infected subjects and providing pre-exposure prophylactic antiretroviral drugs to subjects at increased risks of infection^14^, up to 17 million people in low- and middle-income countries (LMIC) were receiving ART by the end of 2015^15^. In such a context, the emergence and transmission of DRMs is a great concern, especially with the low genetic barrier drugs used in LMICs^8,10–13,16^. To overcome such programmatic challenges, the WHO has developed a surveillance component of HIV drug resistance (HIVDR), which includes in-country monitoring of early warning indicators of HIVDR^17^, assessing the threshold of transmitted or pretreatment DRMs and monitoring acquired HIVDR^16,18^.

As in other SSA countries, ART scale-up is effective in Cameroon, with an increasing national coverage (from 0% in 2003 to 22% in 2014)^19,20^. Therefore, it is critical to monitor HIV-infected Cameroonians for DRMs that could affect ART efficacy. Previous studies of HIV-infected subjects in Cameroon showed treatment failure among some patients on ART, with some of these patients having DRMs, while others did not show any major mutation known to be associated with treatment failure^21^. However, these previous studies of DRMs in Cameroon mainly focused on the viral reverse transcriptase (RT) and protease^21^. Of note, the protease cleaves the 55-kDa viral group specific antigen (Gag) precursor protein (p55) into six structural proteins: the matrix (p17), capsid (p24), spacer peptide-1 (p2), nucleocapsid (NC, p7), spacer peptide-2 (p1) and p6^22,23^. This enzyme also cleaves the 160-kDa GagPol polyprotein precursor into structural proteins and three enzymes: RT, protease, and integrase^22,23^. Protease cleavage occurs at specific cleavage sites on the Gag and GagPol polyproteins^24^, and it has been demonstrated that mutations in Gag cleavage sites can induce resistance to protease inhibitors (PIs)^25–27^ and Nucleoside/Nucleotide Reverse Transcriptase Inhibitors (NRTIs)^28,29^ independently of mutations in the protease, resulting in poor treatment outcomes^27,30^.

The recombinant HIV-1 CRF02_AG is the predominant viral strain circulating in West and Central Africa, including Cameroon (52–80%)^31–34^; but there has been no study, to our knowledge, of Gag DRMs in settings with such HIV molecular epidemiology, and likewise, no study assessing the association between Gag mutations and DRMs in the polymerase, or viremia, and patients’ immunological status in these settings. We therefore sought to ascertain the potential effects of Gag P2/NC cleavage site mutations and polymerase (protease and RT) major DRMs among HIV-infected Cameroonians on treatment outcomes, as well as the possible effects of such interactions on patients’ viral loads and CD4 cell counts, including comparative analyses of CRF02_AG versus non-CRF02_AG.

## Results

### Demographic and clinical characteristics of study subjects

We analyzed plasma samples obtained between 2008 and 2015 from 283 HIV-infected subjects in Yaoundé, Cameroon; 157 samples were from individuals with undetectable viremia, and 126 samples were from subjects with detectable viremia. We successfully amplified and sequenced 113 (89.68%) of the 126 samples from subjects with detectable viremia, and 28 (17.8%) of the 157 samples from subjects with undetectable viremia. Of these 28 samples, we successfully amplified both pol and gag in 8 samples, but could only amplify pol in 4 samples, and gag in 16 samples. Of the total 141 samples amplified and sequenced, 109 (77%) were from ART-naïve subjects. Subjects’ demographics and clinical characteristics are summarized in Table 1.

**Table 1:** Descriptive characteristics of patients included in the study.

Characteristics	Male	Female	*P*-value
N (%)	42 (29.78)	99 (70.21)	
Age (years; mean ± SD)	38.71 ± 9.12	35.23 ± 8.77	0.034
Age range (years)	(18–58)	(20–56)	
Education (years; mean ± SD)	10.44 ± 3.31	9.27 ± 3.92	0.099
Mean CD4 ± SD (cells/µl)	381.6 ± 304.6	322.7 ± 199.7	0.187
CD4 range (cells/µl)	(4–1657)	(12–1233)	
CD4 IQR (cells/µl)	(188–574)	(179.3–416)	
Mean viral load ± SD (log copies/ml)	4.46 ± 1.6	4.11 ± 1.58	0.255
Viral load range (log copies/ml)	(1.6–7.5)	(1.6–7)	
ART Naïve: [N (%)]	35 (83)	74 (74.7)	
**First-line ART [N (%)]**			
3TC-AZT-EFV	3 (7.14)	2 (2.02)	
3TC-AZT-NVP	1 (2.38)	14 (14.14)	
3TC-TDF-EFV	2 (4.76)	2 (2.02)	
3TC-d4T-NVP		2 (2.02)	
3TC-TDF-NVP		2 (2.02)	
**Second line ART [N (%)]**			
2NRTIs-LPV/r	1 (2.38)	3 (3.03)	

N: sample size; SD: standard deviation, ART: antiretroviral therapy; IQR: interquartile range; 3TC: Lamivudine; AZT: Zidovudine, EFV: Efavirenz; TDF: Tenofovir; d4T: Stavudine; NVP: Nevirapine; LPV/r: Lopinavir-Ritonavir; NRTIs: Nucleoside/Nucleotide Reverse Transcriptase Inhibitors.

## Phylogenetic analysis by HIV-1 genomic regions

### Genetic diversity of HIV-1 gag

**Figure 1:**
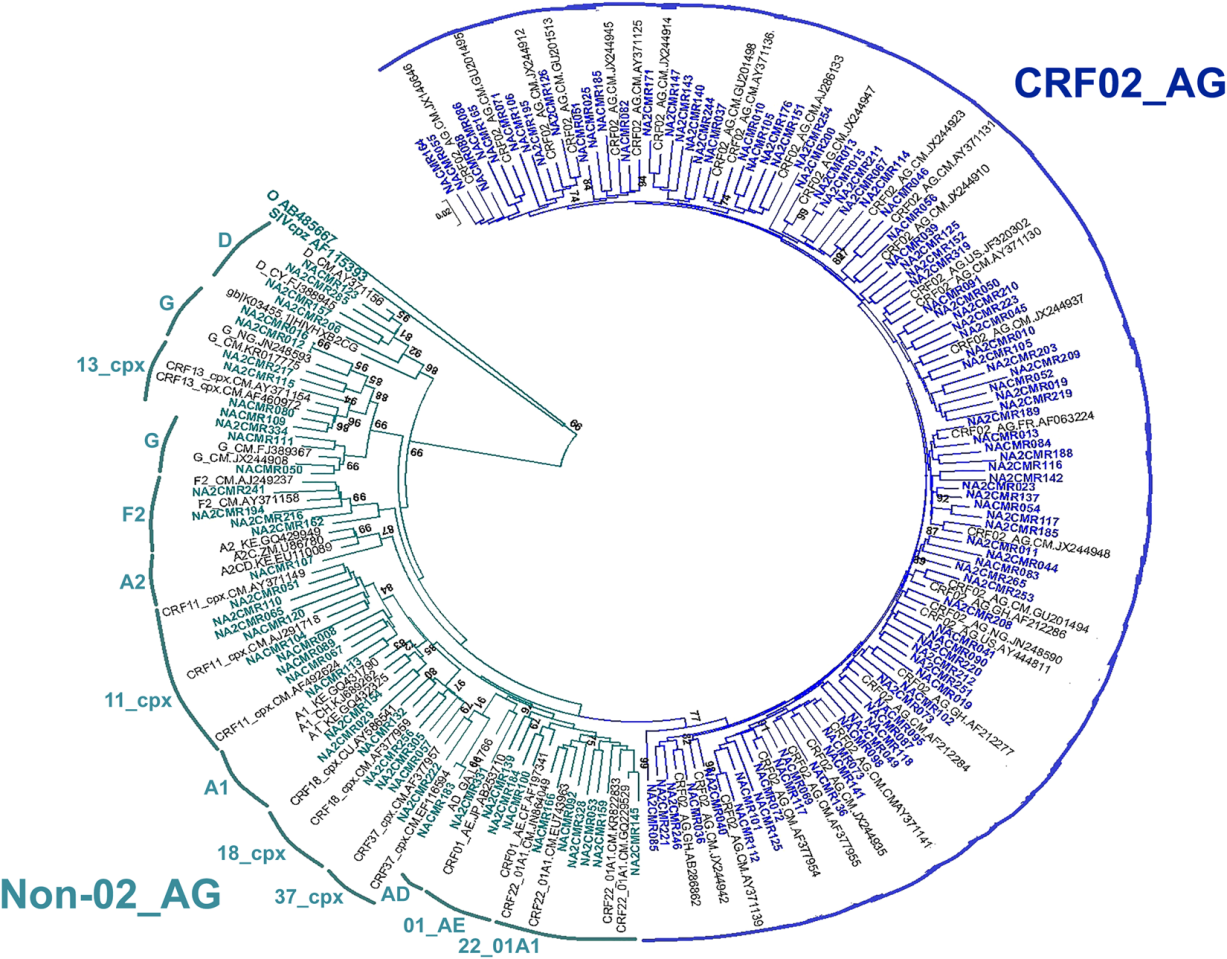
Phylogenetic tree of Cameroon HIV-1 gag sequences. Gag nucleotide sequences of 137 clinical HIV-1 isolates from Cameroon (NACMR or NA2CMR IDs) were aligned using ClustalW, and phylogenetic analysis performed using the neighbor-joining method of MEGA.v.5 software as described in the Methods section. The reference sequences were from the Los Alamos database and included HIV-1 isolates from twelve countries (country letters code precedes reference accession number). Some references have been omitted to enable better visualization of the new Cameroon HIV sequences: CRF02_AG (blue) and non-CRF02_AG (green) subtypes. The Bootstrap value of 1000 replicates of at least 70% was used to determine the HIV-1 subtype. The scale bar represents 2% genetic distance.

We successfully amplified gag sequences from 137 (97.16%) of the 141 samples. Analysis showed that CRF02_AG was the predominant subtype, with 93 subjects (67.15%) harboring HIV-1 CRF02_AG and 44 subjects (32.85%) harboring non-CRF02_AG subtypes [9 (6.56%) CRF11_cpx, 6 (4.37%) CRF22_01A1, 6 (4.37%) subtype G, 4 (2.91%) CRF18_cpx, 4 (2.91%) subtype F2, 4 (2.91%) subtype D, 3 (2.18%) CRF01_AE, 3 (2.18%) CRF13_cpx, 2 (1.45%) CRF37_cpx, 2 (1.45%) subtype A1, 1 (0.72%) CRF35_AD, and 1 (0.72%) subtype A2] (Fig. 1).

### Genetic diversity of HIV-1 pol

**Figure 2:**
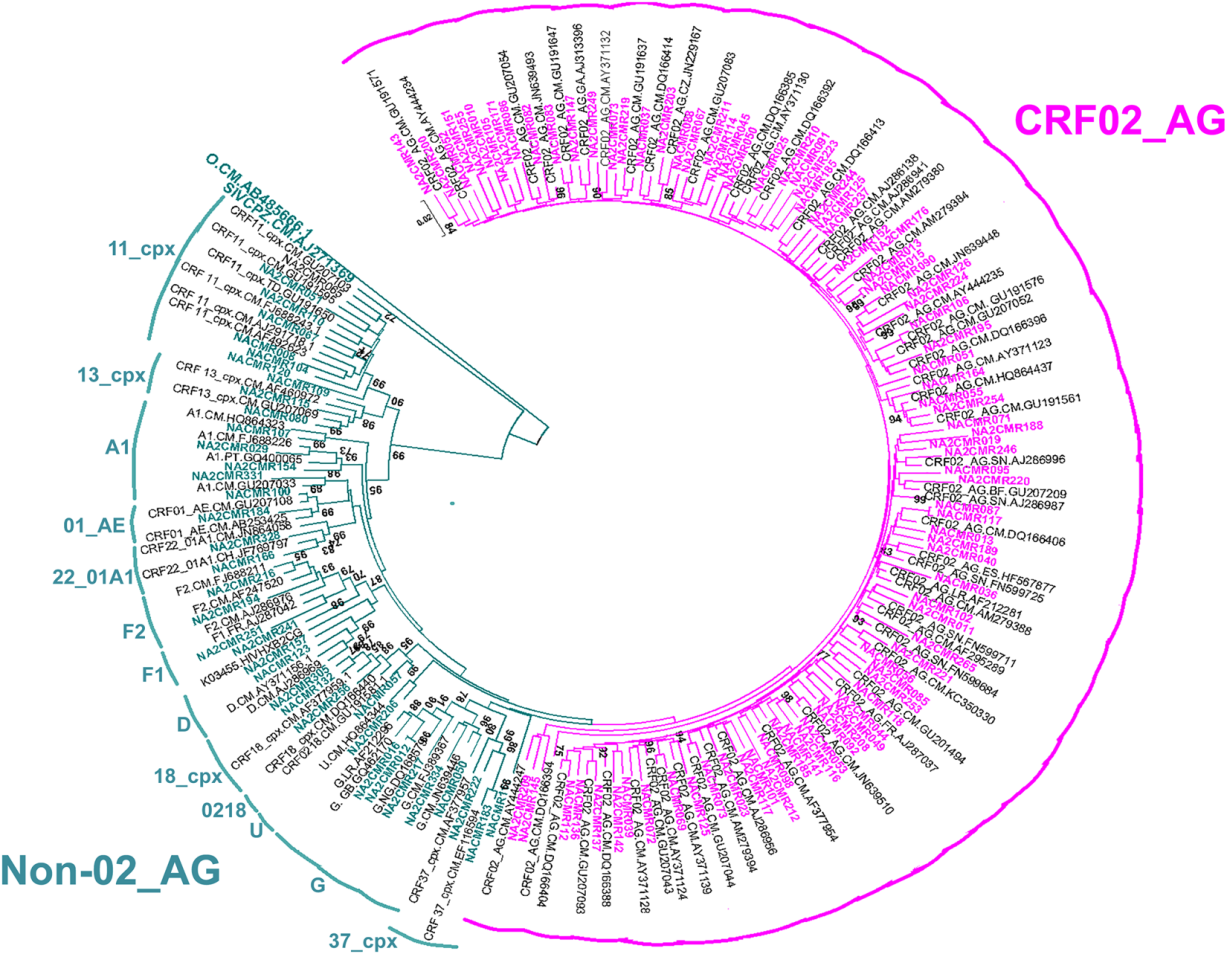
Phylogenetic tree of Cameroon HIV-1 pol sequences. Pol nucleotide sequences of 125 clinical HIV-1 isolates from Cameroon (NACMR or NA2CMR IDs) were aligned using ClustalW, and phylogenetic analysis performed using the neighbor-joining method of MEGA.v.5 software as described in the Methods section. The reference sequences were from the Los Alamos database and included HIV-1 isolates from twelve countries (country letters code precedes reference accession number). Some references have been omitted to enable better visualization of the new Cameroon HIV sequences: CRF02_AG (pink) and non-CRF02_AG (green) subtypes. The Bootstrap value of 1000 replicates of at least 70% was used to determine the HIV-1 subtype. The scale bar represents 2% genetic distance.

We successfully amplified pol sequences (protease: amino acid 1–99; RT: amino acid 1–280) from 125 (88.65%) of the 141 subjects. Analysis of pol sequences showed that HIV-1 CRF02_AG was the most predominant subtype, with 88 subjects (70.4%) harboring CRF02_AG, and 37 subjects (29.6%) with non-CRF02_AG subtypes [7 (5.6%) CRF11_cpx, 5 (4%) subtype G, 4 (3.2%) subtype A1, 3 (2.4%) CRF18_cpx, 3 (2.4%) CRF13_cpx, 2 (1.6%) U (unclassified), 2 (1.6%) subtype D, 2 (1.6%) subtype F1, 2 (1.6%) subtype F2, 2 (1.6%) CRF37_cpx, 2 (1.6%) CRF22_01A1, 2 (1.6%) CRF01_AE, and 1 (0.8%) CRF02_AG/CRF18_cpx] (Fig. 2).

### Overall genetic diversity based on combined gag and pol sequences

**Figure 3:**
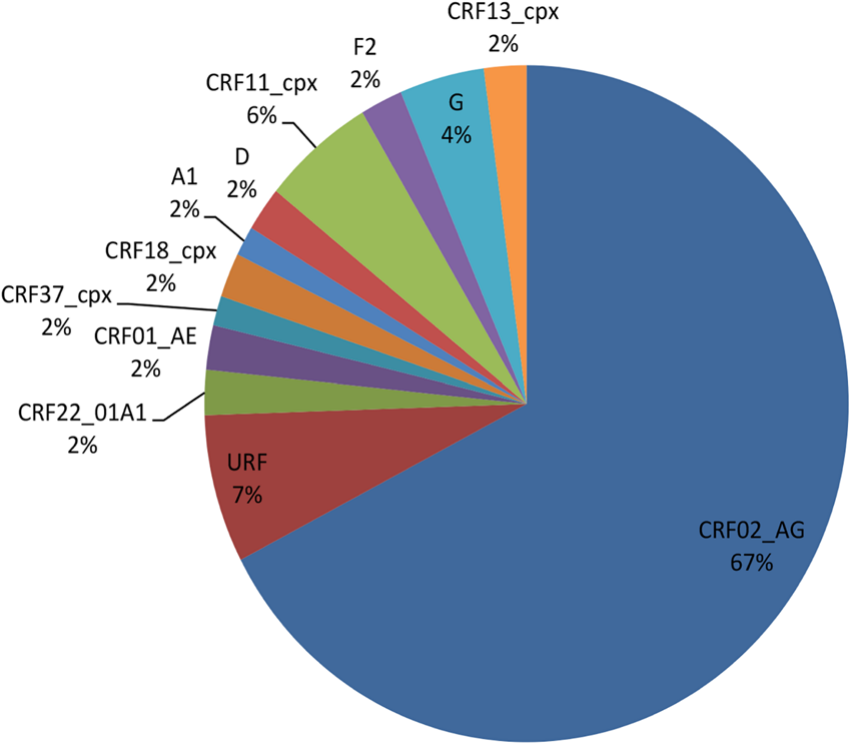
HIV-1 genetic diversity based on pol and gag sequences. Proportions of each HIV-1 subtype and CRFs identified based on the phylogenetic analysis of both gag and pol sequences. CRF, circulating recombinant form; U, unclassified.

Combined analysis of gag and pol sequences from all 141 samples amplified confirmed that CRF02_AG was the most predominant HIV subtype: this strain was present in 95 of the 141 subjects (67.37%), and 46 subjects (32.6%) harbored non-CRF02_AG strains [10 (7%) URFs, 8 (5.67%) CRF11_cpx, 6 (4.2%) subtype G, 3 (2.1%) CRF22_01A1, 3 (2.1%) CRF01_AE, 3 (2.1%) CRF18_cpx, 3 (2.1%) CRF13_cpx, 3 (2.1%) subtype F2, 3 (2.1%) subtype D, 2 (1.4%) subtype A1, and 2 (1.4%) CRF37_cpx (Fig. 3). All the nucleotide sequences analyzed have been validated and submitted to GenBank: gag accession numbers: KX894018 – KX894154; pol accession numbers: KX894155 – KX894279.

### Drug resistance mutations in ART-naïve subjects

Our analysis of pol sequences from 109 treatment-naïve subjects showed 8 subjects (7.3%) (including 6 infected with HIV-1 CRF02_AG) with transmitted DRMs. These transmitted DRMs included major resistance mutations to NRTIs such as M184V, T69D, T215F, K65R, and Y115F; and major resistance mutations to non-Nucleoside/Nucleotide Reverse Transcriptase Inhibitors (NNRTIs) such as Y181C, K103N, P225H, V108I, K101E, Y188L, E138Q, and L100I (Table 2). Four of the 8 treatment-naïve subjects were infected with viruses (HIV-1 CRF02_AG) that had major resistance mutations to both NRTIs and NNRTIs (Table 2). M184V was the most frequent NRTIs resistance mutation and was detected in 3 of the 8 patients (37.5%) (Table 3). Other major NRTIs resistance mutations detected included the multidrug resistance mutation T69D, the thymidine analogue mutation (TAM) T215F, and the non-TAMs K65R and Y115F (Table 3). K103N was the most frequent NNRTIs resistance mutation detected and was present in 4 of the 8 naïve patients (50%) (Table 3). Other major NNRTIs resistance mutations detected included Y181C, V108I, P225H, L100I, K101E, and Y188L (Table 3). No major PI resistance mutation was detected in naïve patients.

**Table 2:** Profile of drug resistance mutations in treatment naïve and subjects on ART.

Sample ID	Treatment status	Resistance mutations			Subtypes
		NRTI	NNRTI	PI	
NACMR091	Naive	M184V	Y181C	—	02_AG
NA2CMR220	Naive	T69D	K103N, P225H	*V11I*, *K20I*	02_AG
NACMR095	Naive	—	V108I	*K20I*	02_AG
NA2CMR305	Naive	—	K101E	*K20I*	18_cpx
NA2CMR331	Naive	—	Y188L	—	URF
NA2CMR116	Naive	M184V, T215F	K103N, E138Q	*K20I*	02_AG
NA2CMR171	Naive	K65R, Y115F, M184V	L100I, K103N	*K20I*	02_AG
NA2CMR189	Naive	—	K103N	*K20I*	02_AG
NA2CMR157	3TC-AZT-NVP	D67N, K70R, M184V, K219Q	K103N	V82L	D
NA2CMR137	3TC-AZT-NVP	M184V, T215F	V106A, P225H	*K20I*	02_AG
NA2CMR176	3TC-AZT-NVP	M184V	Y188L	*K20I*	02_AG
NACMR039	3TC-TDF-LPV/r	K219E	Y181C	*K20I*	02_AG
NA2CMR029	3TC-AZT-NVP	M184V	—	*L10I*	A1
NA2CMR051	3TC-AZT-NVP	D67N, K70R, M184V, K219Q	K103N, H221Y	*L10I*	11_cpx
NA2CMR110	3TC-AZT-EFV	M184V	K103N	—	11_cpx
NA2CMR151	3TC-AZT-NVP	T69N, K70R, M184V, K219Q	Y181C	*L10I*, *K20I*	02_AG
NA2CMR251	3TC-TDF-EFV	M184V	L100I, V106A	*K20I*	URF
NA2CMR249	3TC-AZT-EFV	—	V108I,	*K20I*	02_AG
NA2CMR334	3TC-AZT-NVP	M184V, T215F	V106M	*K20V*	G

AZT: Zidovudine; 3TC: Lamivudine; NVP: Nevirapine; TDF: Tenovofir; LPV/r: Lopinavir/ritonavir; NRTI: Nucleoside/Nucleotide Reverse Transcriptase Inhibitors; NNRTI: Non-Nucleoside/Nucleotide Reverse Transcriptase Inhibitor; PI: Protease Inhibitor (minor resistance mutations are in italic).

**Table 3:** Resistance mutations, viral subtypes, and ART.

ART	Mutations	Treatment status		Subtypes		Total n (%)
		ART (n)	Naïve (n)	02_AG (n)	Non-02_AG (n)	
**NRTI**	M184V	9	3	6	5	11(58)
	T69D	1	1	2	—	2(11)
	K65R	—	1	1	—	1(5)
	Y115F	—	1	1	—	1(5)
	K219E/Q	4	—	2	2	4(21)
	D67N	2	—	—	2	2(11)
	K70R	3	—	1	2	3(16)
	T215F	2	1	2	1	3(16)
**NNRTI**	Y181C	2	1	3	—	3(12)
	V106M/A	3	—	1	2	5(20)
	V108I	1	1	2	—	2(8)
	K103N	3	4	4	3	7(28)
	P225H	1	1	2	—	2(8)
	L100I	1	1	1	1	2(8)
	K101E	—	1	—	1	1(4)
	Y188L	1	1	1	1	2(8)
**PI**	V82L	—	1	—	1	1(4)

NRTI: Nucleoside/Nucleotide Reverse Transcriptase Inhibitors; NNRTI: Non- Nucleoside/Nucleotide Reverse Transcriptase Inhibitor; PI: Protease Inhibitor; n: sample size; ART: antiretroviral therapy; 02_AG: HIV-1 CRF02_AG.

### Drug resistance mutations in subjects on ART

Of the 32 samples successfully amplified and sequenced from treatment-experienced patients, 28 (87.5%) were from subjects on first-line ART regimen [two NRTIs (lamivudine (3TC) + zidovudine (AZT) or 3TC + tenofovir (TDF)), and a NNRTI: nevirapine (NVP) or efavirenz (EFV)], and 4 (12.5%) were from subjects on second-line regimen [two NRTIs + lopinavir-ritonavir (LPV/r)] (Tables 1 and 2). Analysis of pol sequences showed that 11 of these 32 subjects (34.3%) were infected with viruses harboring major DRMs, including major resistance mutations to NRTIs such as D67N, K70R, M184V, K219Q/E, T215F, and T69N; and major resistance mutations to NNRTIs such as Y181C, K103N, V106A/M, P225H, Y188L, H221Y, V108I, L100I (Tables 2 and 3). Six of the 11 subjects with major DRMs harbored viruses with the thymidine analogue mutations (TAMs) D67N, K70R, K219E/Q, and T215F (Tables 2 and 3). Nine of these 11 subjects harbored viruses with major resistance mutations to both NRTIs and NNRTIs (Table 2). One subject (NA2CMR157) harbored viruses that had major resistance mutations to NRTIs, NNRTIs, and PIs (Table 2). None of the 4 subjects on second line ART harbored viruses with major resistance mutation to PIs, but one of these subjects (NACMR039) harbored viruses with major resistance mutations to both NRTIs and NNRTIs, and a minor/secondary resistance mutation to PIs (K20I) (Table 2). Four subjects on ART also had viruses harboring the NNRTIs secondary mutations V179D, A98G, E138A, V179E, and F227L, including subject NA2CMR151 who had both A98G and the major NNRTI resistance mutation Y181C. Most of the subjects with major resistance mutation to NRTIs and/or NNRTIs harbored viruses that also had secondary resistance mutations to PIs: L10I, V11I, K20V, and K20I (Table 2). Overall, M184V and K103N were the most prevalent NRTIs and NNRTIs transmitted or acquired DRMs, both for subjects infected with HIV-1 CRF02_AG and those infected with non-CRF02_AG subtypes (Table 3).

### Effects of viral subtypes and drug resistance mutations on patients’ viral load and CD4+ cell count

Analysis of viral and immunological parameters showed no differences in CD4+ cell counts of treatment-naïve and subjects on ART, either for subjects infected with CRF02_AG, or non-CRF02_AG viruses (Table 4). There was no difference in CD4+ cell counts of treatment-naïve subjects with NNRTIs resistance mutations, compared to subjects on ART that had NNRTIs resistance mutations (Table 4). However, CD4+ cell counts were significantly lower in treatment-naïve subjects with NRTIs resistance mutations (148.3 ± 79.5 cells/µl), compared to subjects on ART that had NRTIs resistance mutations (291.4 ± 108 cells/µl) (Table 4, P = 0.042).

**Table 4:** Effects of drug resistance mutations and viral subtypes on CD4 cells counts and viral load.

Parameters	Status	Subtypes		DRMs	
		02_AG	Non-02_AG	NRTIs	NNRTIs
Mean CD4 ± SD (Cells/µl)		(n = 95)	(n = 46)	(n = 14)	(n = 18)
	ART (n = 18)	350.4 ± 276.7	321.4 ± 141.2	291.4 ± 108	336.2 ± 343
	Naïve (n = 74)	353.9 ± 256.3	313.1 ± 191.8	148.3 ± 79.5	260.0 ± 246.8
	***P*** **-value**	0.959	0.886	0.042	0.605
Mean VL ± SD (log copies/ml)	ART (n = 18)	3.31 ± 1.34	2.8 ± 1.56	2.7 ± 1.4	3.13 ± 1.5
	Naïve (n = 74)	4.48 ± 1.59	4.6 ± 1.2	3.77 ± 1.52	4.57 ± 1.64
	***P*** **-value**	0.0049	0.0002	0.256	0.07

VL: Viral Load; DRMs: Drug Resistance Mutation; SD: standard deviation; 02_AG: HIV-1 CRF02_AG; n: sample size NRTI: Nucleoside/Nucleotide Reverse Transcriptase Inhibitors; NNRTI: Non- Nucleoside/Nucleotide Reverse Transcriptase Inhibitor; ART: antiretroviral therapy.

Treatment-naïve subjects infected with HIV-1 CRF02_AG had higher viral loads (4.48 ± 1.59 log copies/ml) than subjects on ART infected with HIV-1 CRF02_AG (3.31 ± 1.34 log copies/ml) (Table 4, P = 0.0049). Treatment-naïve subjects infected with non-CRF02_AG viruses had higher viral loads (4.6 ± 1.2 log copies/ml) than non- CRF02_AG infected subjects on ART (2.8 ± 1.56 log copies/ml) (Table 4, P = 0.0002). There was no significant difference in viral loads of treatment-naïve subjects with NRTIs DRMs, compared to infected subjects on ART that had NRTIs DRMs (Table 4, P = 0.256). However, treatment-naïve subjects with NNRTIs DRMs tended to have higher viral loads (4.57 ± 1.64 log copies/ml) than infected subjects on ART with NNRTIs DRMs (3.13 ± 1.5 log copies/ml) (Table 4, P = 0.07).

### Gag P2/NC cleavage site mutations

**Figure 4:**
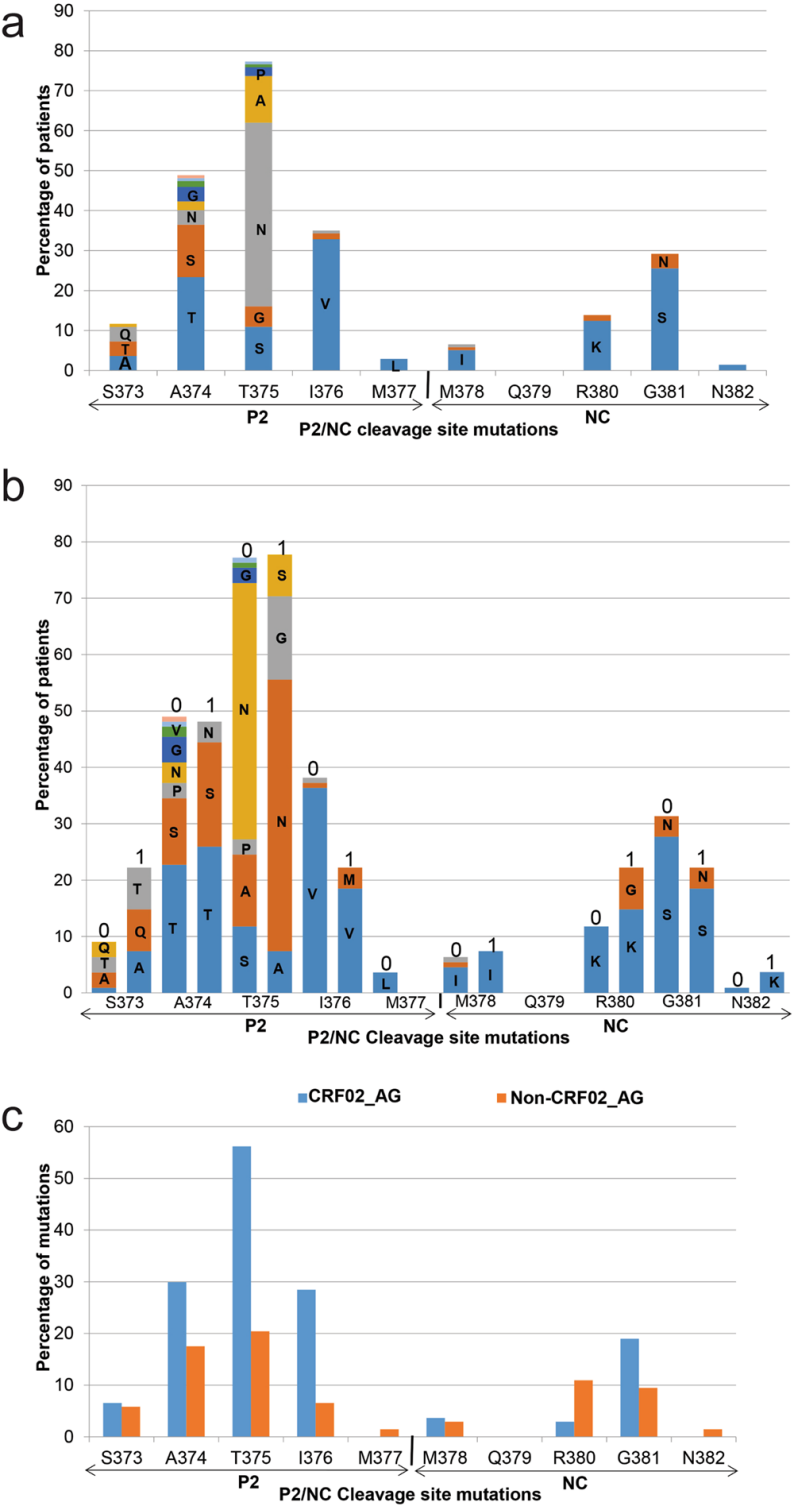
Frequency of mutations in P2/NC cleavage site. Data show the mutations in the Gag P2/NC cleavage site, and the proportion of patients with each mutation (panel a), the proportion of treatment-naïve (0) and subjects on ART (1) having each mutation (panel b); and the frequency of each P2/NC cleavage site mutation in subjects infected with HIV-1 CRF02_AG and non-CRF02_AG subtypes (panel c).

Among the 141 samples successfully amplified and sequenced, 137 (93.19%) were HIV-1 gag sequences, of which 27 (19.70%) were from subjects on ART and 92 (67.15%) were HIV-1 CRF02_AG. Analysis of the P2/NC Gag cleavage site showed the following mutations: S373A/T/Q/P, A374T/S/N/G/P/V/H/Q, T375S/G/N/A/P/H/I, I376V/M/A, M377L, M378I/L/V, R380K/G, G381S/N, and N382K (Fig. 4a). No mutation was found in the Q379 residue. Of the subjects analyzed, 16 (11.67%), 8 (5.83%), and 1 (0.72%) harbored viruses with T375A, S373Q, and S373P mutations respectively in the P2/NC cleavage site (Fig. 4a). Three subjects had mutations at both HIV-1 Pol and Gag P2/NC cleavage sites. Subject NA2CMR151 harbored viruses with both the Gag cleavage site mutations S373Q, T375G, I376V, G381S; the major NRTIs resistance mutations T69N, K70R, M184V, K219Q; the major NNRTIs resistance mutation Y181C, and the secondary NNRTIs resistance mutations A98G. Subject NA2CMR176 harbored viruses with both Gag cleavage site mutations A374T, T375G, I376V, G381S, the major NRTIs resistance mutations M184V, and the major NNRTIs resistance mutation Y188L. Subject NACMR050 harbored viruses with both Gag cleavage site mutation T375A and the secondary NNRTI resistance mutation V179E.

### Gag P2/NC cleavage site mutations in treatment-experienced and naïve patients

P2/NC cleavage site mutations often occurred at similar position and involved similar amino acid in sequences from both treatment-naïve and subjects on ART, although both groups often showed differences in the percentage of subjects with each specific mutation (Fig. 4b). Naïve subjects showed some mutations in the P2/NC cleavage site that were absent in ART-experienced patients, including S373P, A374G/V/H/Q, T375P/H/I, I376A, M377L, and M378L/V. All treated subjects with Gag P2/NC cleavage site mutations were on first line ART regimen. The R380G mutation was present only in subjects on ART. Further analysis to identify subjects with more than two mutations in the Gag P2/NC cleavage site showed that Gag sequences from 12 of the 137 (8.75%) patients harbored viruses with 3 to 4 mutations in the P2/NC cleavage site (Table 5). Eight were treatment-naïve and 4 were on 3TC-AZT-NVP (Table 5). Eleven of these 12 subjects with ≥3 mutations in the Gag P2/NC cleavage site also had secondary PIs resistance mutations, including 10 subjects with K20I (Table 5).

**Table 5:** Subjects with at least 3 mutations in the Gag P2/NC cleavage site.

Sample ID	P2/NC	PIs DRMs		
	Cleavage site mutations	AMs	Treatment	Subtypes
NA2CMR010	T375A, I376V, G381S	K20I	3TC-AZT-NVP	02_AG
NA2CMR013	T375A, I376V, G381S	K20I	Naïve	02_AG
NA2CMR115	A374S, T375S, R380K, G381S	K20I, K43T	Naïve	13_cpx
NA2CMR147	A374N, T375S, I376V, G381S	K20I, L10V	Naïve	02_AG
NA2CMR151	S373Q, T375G, I376V, G381S	K20I, L10I	3TC-AZT-NVP	02_AG
NA2CMR176	A374T, T375G, I376V, G381S	K20I	3TC-AZT-NVP	02_AG
NA2CMR216	S373A, A374S, R380K, G381S	L10V	3TC-AZT-NVP	F2
NACMR071	A374S, T375N, I376V, G381S	K20I	Naïve	02_AG
NACMR165	S373Q, A374T, I376V	*	Naïve	02_AG
NACMR052	S373T, A374G, I376V, G381S	K20I, E35G	Naïve	02_AG
NACMR056	S373A, A374S, T375A	K20I	Naïve	02_AG
NACMR086	S373Q, A374T, T375S, I376V	K20I, L33F	Naïve	02_AG

PIs: protease inhibitors; DRMs: drug resistance mutations; AMs: accessory (secondary) mutations; AZT: zidovudine; 3TC: lamivudine; NVP: nevirapine; 02_AG: HIV-1 CRF02_AG; 13_cpx: CRF13_cpx. *Sample not amplified.

### Gag P2/NC cleavage site mutations in HIV-1 CRF02_AG and non-CRF02_AG subtypes

Ten of the 12 subjects with ≥3 mutations in the Gag P2/NC cleavage site were infected with HIV-1 CRF02_AG (Table 5). Subtype analysis showed higher percentage of mutations in the P2/NC cleavage site amino acid residues S373, A374, T375, I376, M378 and G381 in Gag sequences from subjects harboring HIV-1 CRF02_AG compared to subjects infected with Non-CRF02_AG viruses (Fig. 4c). HIV-1 CRF02_AG isolates showed lower percentage of mutations in the P2/NC cleavage site residues M378 and 380 and no mutations in the gag residues M377 and N382 (Fig. 4c). Mutations at residues 377 and 382 were observed only in non-CRF02_AG isolates and these non-CRF02_AG subtypes also showed a higher percentage of mutations at residue R380 (Fig. 4c). Both HIV-1 CRF02_AG and Non-CRF02_AG isolates showed no mutation at residue Q379.

### Gag P2/NC cleavage site mutations, viral loads and CD4 cell counts

We performed additional analyses to determine whether mutations in the Gag P2/NC cleavage site had any effects on viral loads and CD4 cell counts. Of the 137 subjects with Gag sequences, 11 had 3 or more mutations in the Gag P2/NC cleavage site while 126 had less than 3 mutations in the P2/NC cleavage site. There was no significant difference in the viral loads of subjects with ≥3 mutations and subjects with less than 3 mutations in the P2/NC cleavage site (Table 6). Analysis of all subjects with a mutation in the Gag P2/NC cleavage site showed higher viral loads in treatment-naïve subjects compared to subjects on ART (Table 6, P = 0.0005). There was no difference in CD4 cell counts of subjects with ≥3 mutations and those with less than 3 mutations in the P2/NC cleavage site; and no difference in CD4 cells counts of subjects with Gag P2/NC mutations that were treatment-naïve or were on ART (Table 6).

**Table 6:** Effects of Gag P2/NC mutations on viral loads and CD4 cell counts.

Status	Parameters	
	VL (log copies/ml)	CD4 count (cells/µl)
≥3 mutations	3.53 ± 1.46 (n = 11)	339.9 ± 262.9 (n = 11)
<3 mutations	5.27 ± 1.6 (n = 126)	339.7 ± 233.7 (n = 124)
***P-value***	0.137	0.9976
ART (Mean ± SD)	3.338 ± 1.5 (n = 27)	300.4 ± 158.2 (n = 27)
Naïve (Mean ± SD)	4.5 ± 1.5 (n = 106)	343.9 ± 238.5 (n = 104)
***P-value***	0.0005	0.3712

VL: viral loads, SD: standard deviation; ART: antiretroviral therapy; n: sample size.

## Discussion

Mutations on the HIV genome can result in the emergence of viruses that are resistant to current ART drugs and treatment failure^27,30^. Previous studies in Cameroon showed treatment failure among HIV-infected subjects on ART, with some associated with DRMs^21^. However, these studies only analyzed the polymerase and none examined mutations on Gag sequences. The viral protease cleaves the Gag polyprotein precursors into six structural proteins (p17, p24, p2, NC/p7, SP2/p1, and p6)^22,23^, and cleaves the GagPol polyprotein precursor into structural proteins and the enzymes RT, protease, and integrase^22,23^. Studies of HIV-1 subtype B showed that mutations on Gag or near the protease cleavage sites can induce resistance to antiretroviral drugs and treatment failure, independent of mutations in other segments of the viral genome^25–29^, and directly contribute to resistance to PIs^25–27,35^. This is, to our knowledge, the first study to examine DRMs in Gag sequences of HIV-1 CRF02_AG, in correlation with DRMs in the RT and protease, viremia, and immune function.

Gag sequences from 12 of the 137 subjects analyzed in this study, 10 of whom were infected with HIV-1 CRF02_AG, showed ≥3 mutations in the Gag P2/NC cleavage site, including S373Q/T/A, A374T/S/G/N, T375S/A/N/G, I376V, and G381S. These mutations have been associated with drug resistance and treatment failures in subjects infected with other viral subtypes^27,30^. Studies of HIV-1 subtype B showed that mutations in the Gag NC-SP2-P6 region occurred prior to mutations in the protease, and contributed to decreased viral susceptibility to antiretroviral drugs and resistance to PIs^25,36^. Other studies of subjects infected with HIV-1 subtypes B, A, C, G, and D^30,37–39^, showed that mutations in Gag associated with resistance to PIs (LPV, and SQV/r) and treatment failure included mutations at amino acid residues 373 (including S373Q/P), 374, 375, and 378 at the P2/NC cleavage site. These mutations were observed before and after treatment failure, and resulted in the emergence of genetically distinct viruses at the time of treatment failure^38^.

Viral genotype could also influence mutations and treatment efficacy. A study of patients infected with subtype-B and non-B HIV-1 on LPV/r monotherapy showed that at baseline, non-B subtypes were significantly more likely to harbor mutations, and the presence of more than 2 mutations in the P2/NC cleavage site at baseline predicted virologic failure^40^. This suggests that the 12 subjects identified in our current study with 3 to 4 mutations in the Gag P2/NC cleavage site could be more susceptible to treatment failure if given PIs. These subjects also had secondary mutations in the protease, including K20I that was previously shown to be associated with failure of PIs-based ART when simultaneously present with mutations in the Gag amino acids residues 373, 374, and/or 375^30,38^.

In our current study, mutations in the P2/NC cleavage site did not significantly affect viral loads or CD4 cell counts. This could result from mutations in Gag being more likely to affect PIs efficacy, whereas only 4 of the 141 subjects were on PIs, and none of the subjects with ≥3 mutations in the Gag P2/NC cleavage site had been on PIs. In fact, it has been shown that mutations in the Gag cleavage sites, as well as mutations outside the cleavage sites, change the structure of the Gag substrate, and this can reduce the PIs’ affinity to the Gag-binding cleft and render PIs inefficient, resulting in restoration of the viral fitness and treatment failure^41,42^. In addition to affecting PIs’ efficacy, mutations in the Gag cleavage sites can increase viral infectivity and resistance to NRTIs^29^.

In the current study, analysis of RT and protease sequences from 109 HIV-infected and treatment-naïve Cameroonians showed 8 (7.3%) with transmitted DRMs, including major resistance mutations to NRTIs (M184V and T215F) and NNRTIs (L100I, Y181C, K103N, V108I, and Y188L). Previous analysis of pol sequences from 116^43^ and 216^44^ treatment-naïve subjects in Cameroon showed a 13.9%^43^ and 8.2%^44^ rates of transmitted DRMs. Our data confirm these previous findings and show ongoing transmission of viral populations with DRMs in Cameroon.

Analysis of Pol sequences from the 32 patients on ART also showed 11 (34.3%) with major DRMs. In addition to the resistance mutations to NRTIs (M184V and the TAM T215F) and NNRTIs (L100I, Y181C, K103N, V108I, and Y188L) observed in naïve subjects, these subjects on ART also had the TAMs D67N, K70R, and K219Q; and 9 of those 11 subjects had major resistance mutations to both NRTIs and NNRTIs. These higher proportions of DRMs in subjects on ART confirm increased prevalence of acquired resistance mutations to NRTIs and NNRTIs in Cameroon. Increased prevalence of acquired DRMs and transmission of drug resistant mutants to other Cameroonians could result in increased risk of treatment failure and pose a major challenge to the local and global efforts against HIV/AIDS.

M184V was the most common NRTIs resistant mutation in both treatment-naïve and subjects on ART. M184V mutation reduces the incorporation of nucleotide analogs into DNA, resulting in increased resistance to 3TC and emtricitabine^45–47^. The TAMs T215F, D67N, K70R, and K219Q induce resistance to the thymidine analogues AZT, stavudine, and other NRTIs by enhancing ATP-mediated excision and hydrolytic removal of the drug incorporated into viral DNA, thereby unblocking the viral DNA chain and enabling continuation of viral replication^48,49^.

The major NNRTIs resistance mutations identified in both treatment-naïve and subjects on ART included L100I, Y181C, K103N, V108I, and Y188L. These mutations are known to decrease the binding affinity of EFV and NVP to the viral target, resulting in resistance to these antiretroviral drugs and increased risk of virologic failure^47,50,51^. All subjects on ART in our study whose viral sequences showed major resistance mutations to NRTIs, NNRTIs, or more than 2 mutations in the Gag P2/NC cleavage site were on regimens including 3TC; and most of these subjects were on regimens including AZT, NVP, or EFV, suggesting a potential risk of future virologic failure in those patients. This risk would further increase in subjects harboring viruses with multiple DRMs. In fact, 5 of the 8 treatment-naïve subjects with transmitted DRMs and 9 of the 11 subjects with acquired DRMs had major resistance mutations to both NRTIs and NNRTIs. One subject had major resistance mutations to NRTIs, NNRTIs, and PIs, and two subjects had major resistance mutations to NRTIs, NNRTIs, and 4 mutations in the Gag P2/NC cleavage site. These multiple DRMs could increase the risk of treatment failure. In fact, analysis of pol sequences from 216 HIV-infected subjects starting ART showed that 80% of patients failing first line ART harbored viruses with at least 1 resistance mutation to two antiretroviral drug classes, and 36% of those failing second line ART harbored viruses with at least 1 resistance mutation to three antiretroviral drug classes^44^.

Despite this risk of drug resistance, ART is necessary and is beneficial for all infected subjects. Our data showed overall lower viral loads in subjects on ART, better immune recovery with significantly higher CD4 counts in subjects with major resistance mutations to NRTIs who were on ART, compared to treatment-naïve subjects with major resistance mutations to NRTIs. Our data also showed better viral control and borderline significant lower viral load in subjects with major resistance mutations to NNRTIs who were on ART, compared to treatment-naïve subjects with major resistance mutations to NNRTIs. One subject had major resistance mutations to NRTIs, NNRTIs and PIs; 9 subjects had major resistance mutations to both NRTIs and NNRTIs; two subjects had major resistance mutations to NRTIs, NNRTIs, and 4 mutations in the Gag P2/NC cleavage site. It is possible that such multiple DRMs could negatively impact treatment efficacy, viremia, and immune recovery, but studies with a larger sample size would be required to assess these effects. It is also likely that multiple mutations in the Gag cleavage sites, as shown in 12 subjects with 3 to 4 mutations in the Gag P2/NC cleavage sites, could affect the efficacy of PIs-based ART.

### Conclusions and recommendations

In summary, our data confirmed previous findings of HIV-1 CRF02_AG predominance in Cameroon (52–80%)^32–34^ and showed the presence of transmitted (7.3%) and acquired (34.3%) resistance mutations to both NRTIs and NNRTIs. Our data also showed mutations in Gag P2/NC cleavages sites, with 12 subjects [8 treatment-naïve and 4 on 3TC-AZT-NVP] showing 3 to 4 mutations in the Gag P2/NC cleavage site. These results have clinical implications because the Gag P2/NC cleavage site mutations identified have been associated with resistance to PIs in subjects infected with HIV-1 subtype-B^25–27,35^. The presence of these Gag mutations in treatment-naïve and subjects not previously exposed to PIs could result in increased risk of virologic failure with use of PIs-based ART. Only 4 subjects in this study were on PIs-based ART, but with the potential increased use of second line regimen in Cameroon, subjects should be monitored for mutations in Gag cleavage sites that could affect the efficacy of PIs-based ART.

### Study limitations

We do not know whether the small numbers of non-CRF02_AG subtypes played a role in the lower frequency of mutations observed in non-CRF02_AG, compared to HIV-1 CRF02_AG sequences. Like in other countries in West and Central Africa, the molecular epidemiology of HIV in Cameroon is characterized by the predominance of HIV-1 CRF02_AG (over 67% of our samples), and despite the high viral genetic polymorphism, the frequency of the other 10 HIV-1 subtypes and URFs identified was low (1 to 7%), with some subtypes identified in only 1 or 2 subjects. Additional non-parametric regression analyses could have identified potential correlation between DRMs and individual subtypes, gender, age, and ART regimens. But with small sample sizes for non-CRF02_AG subtypes, we could not obtain sufficient statistical power for such analyses. For similar reasons, we were not able to analyze the correlation between Gag P2/NC cleavage site mutations and Pol DRMs. In fact, only 3 subjects with Gag P2/NC cleavage site mutations also had mutations in the Pol region. Future studies with larger sample size for subtypes that characterize the HIV molecular epidemiology in Cameroon would enable such correlation analyses.

The present study focused on Gag P2/NC, a primary cleavage site that play a critical role in virions production and maturation; there are four other cleavage sites on Gag and six cleavage sites on the GagPol polyproteins that are also involved in virions production, maturation, and fitness^26,52–54^. Mutations in those sites could also result in drug resistance and virologic failure for subjects on PIs-based therapy^26,30,53,55,56^. Our subsequent studies will analyze these Gag and GagPol cleavage sites for the presence of DRMs and their impact on treatment outcomes. The presence of a major PI resistance mutation in only one subject in our study is likely due non-exposure to this drug class (only about 3% had been on PIs-based therapy). With the increasing use of second line regimens and PIs-based ART in SSA, studies of DRMs on the Gag and GagPol cleavage sites are important; such mutations would be of clinical and therapeutic consequences.

## Materials and Methods

### Study design, population, and ethical considerations

A cross-sectional analysis was conducted on plasma samples from 283 HIV-1 infected individuals in Yaoundé, Cameroon, between 2008 and 2015. These samples were collected as part of an ongoing project aimed at analyzing the influence of HIV genetic diversity on viral neuropathogenesis. This study was performed in accordance with guidelines of the Helsinki Declaration and was approved by the Cameroon National Ethics Committee, as well as the Institutional Review Board of the University of Nebraska Medical Center. Written informed consent was obtained from all participants and data were processed using unique identifiers to ensure confidentiality.

### HIV serology, CD4 cell counts, and viral load

Sample collection and analyses were performed in the Hematology laboratory of the Yaoundé University Teaching Hospital, Cameroon. Venous blood samples were collected and stored at room temperature in the Hematology laboratory, and analyses performed within 6 hours of blood collection. The HIV status of each participant was determined using the Alere Determine HIV-1/2 antigen/antibodies Combo (Jouy-En-Josas, France), and the Murex HIV antigen/antibody Combination ELISA (Abbott Diagnostics, Chicago, IL, USA), according to the manufacturer’s instructions. Each batch of reagents was quality controlled with known samples before used. A participant was considered HIV-positive if he/she tested positive for the two tests, HIV-negative if non-reactive for both tests and discordant if positive for only one test. No discordant result was observed in this study.

CD4 T-lymphocyte count was quantified by flow cytometry, using a Fluorescence Activated Cell Sorting (FACS) Count Instrumentation System, BD FACSCount, according to the manufacturer’s instructions (BD Biosciences, San Jose, CA, USA). The FACS instrument was calibrated and quality control tested before each experiment. Plasma samples were stored at −70 °C or lower. For viral load quantification, HIV RNA copies number in each plasma sample was quantified by reverse transcription polymerase chain reaction (RT-PCR), using Amplicor HIV-1 Monitor Test (Roche Diagnostic Systems, Pleasanton, CA), according to the manufacturer’s protocol. The Amplicor HIV-1 Monitor Test detection limit was 50 viral copies/ml.

### RNA extraction, cDNA synthesis and polymerase chain reaction

Plasma samples were shipped on dry ice (−70 °C) to the University of Nebraska Medical Center, where all other analyses were performed. Viral RNA was extracted from plasma samples using the QIAmp viral RNA Mini kit (Qiagen Inc., Valencia, CA, USA) according to the manufacturer’s protocol; 350 to 1200 ng RNA were reverse transcribed and amplified using a nested PCR with SuperScript One-Step RT-PCR reverse transcriptase and Platinum Taq DNA polymerase (Life Technologies, Carlsbad, CA, USA), according to the manufacturer’s instructions. The 1200 nucleotides polymerase (pol) gene was amplified in a 50 μL reaction volume containing 7.5 pmoles of each of the following forward (5′-GACAGGCTAATTTTTTAGGG-3′; 2078–2094 bp of HXB2) and reverse (5′-TTTCCC CATATTACTATGCTT-3′; 3700–3683 bp of HXB2) primers. Pol RT-PCR was performed using the following conditions: 50 °C, 30 min; 94 °C, 2 min; 40 cycles of 95 °C, 30 s; 53 °C, 30 s; 72 °C, 1 min 30 s; and a final extension step at 72 °C, 10 min. The 700 nucleotides gag gene was amplified using the following forward (5′-TCACCTAGAACTTTGAATGCATGGG-3′; 1234–1255 bp of HXB2), and reverse (5′-CTAATACTGTATCATCTGCTCCTGT-3′; 2349–2328 bp of HXB2) primers; and gag RT-PCR performed using the following conditions: 50 °C, 30 min; 94 °C, 2 min; 40 cycles of 94 °C, 30 s; 50 °C, 30 s; 68 °C, 1 min 30 s; and a final extension step at 68 °C, 7 min.

Five microliters of each RT-PCR reaction product was used in a second/nested PCR, in a 50 μL reaction volume containing 7.5 pmoles each of the following forward (5′-GACAGGCTAATTTTT TAGGG-3′; 2078–2094 bp of HXB2) and reverse (5′-GGC TCT TGA TAA ATT TGA TAT GT-3′; 3580–3561 bp of HXB2) primers for the pol gene, under the following conditions: 93 °C, 12 min; 40 cycles of 94 °C, 30 s; 53 °C, 30 s; 72 °C, 2 min; and a final extension step at 72 °C, 10 min. Similarly, gag nested PCR was performed in a 50 μL reaction volume containing 7.5 pmoles each of the forward (5′-AAAGATGGATAATCCTGG G-3′; 1580–1595 bp of HXB2) and reverse (5′-TCCACATTTCCAACAGCC CTTTTT-3′; 2037–2017 bp of HXB2) primers, under the following conditions: 94 °C, 2 min; 40 cycles of 94 °C, 30 s; 50 °C, 30 s; 72 °C, 1 min; and a final extension step at 72 °C, 7 min^44^. Amplicons were detected by electrophoresis on a 1% agarose gel and visualized by ethidium bromide staining under ultraviolet light.

### DNA sequencing and phylogenetic analysis

Nucleotide sequences were obtained by direct sequencing of the PCR products. Briefly, amplicons were purified using Amicon Microcon Ultra-pure kit (Centrifugal Filters Devices, Millipore, Billerica, MA, USA) according to the manufacturers′ instructions. DNA sequencing was performed at the University of Nebraska Medical Center High-Throughput DNA Sequencing and Genotyping Core Facility, using a 20 μL reaction mix containing 62 ng of the purified gag PCR products, each RT-PCR and nested PCR gag primers (12.8 pmoles forward primer or 12.8 pmoles reverse primer) and the Big-Dye chemistry method (Perkin-Elmer, Austin, TX, USA). Pol sequencing was performed using the following eight overlapping sequence-specific primers (5′-AGCAGACCAGAGCCAACAGC-3′; 2143–2159 bp of HXB2); (5′-ATTTTCCCTTCCTTTTCCATTTC-3′; 2686–2667 bp of HXB2); (5′-TTGTACAGAAATGGAAAAGGAAGG-3′; 2663–2683 bp of HXB2); (5′-TTTGTTCTATGCTGCCCTATTTCT-3′; 3148–3128 bp of HXB2); (5′-GGCAGCATAGAACAAAAATAGAGG-3′; 3139–3159 bp of HXB2); (5′-CAGGAATGGATGGCCCAAAA-3′; 2593–2609 bp of HXB2); (5′-GCTTCCACAGGGATGGAAA-3′; 2996–3011 bp of HXB2); (5′-CCATCCATTCCTGGCTTTAAT-3′; 2599–2582 bp of HXB2). Each of these primers was used at 12.8 pmoles in a 20 μL reaction mix including 130 ng of the purified pol PCR product (protease: amino acid 1–99; and reverse transcriptase: amino acid 1–280). Pol sequencing was performed using the Big-Dye chemistry method (Perkin-Elmer).

Capillary electrophoresis was performed using an Applied Biosystems 3730 DNA sequencer (Applied Biosystems, Tokyo, Japan) and sequences were loaded and assembled into Pregap4 v.1.5 software to generate contigs^57^. Nucleotide sequences were aligned with subtype/CRFs reference sequences from the Los Alamos National Laboratory (LANL) database using the CLUSTAL.W integrated into Bioedit.7.2.5 software^58,59^. Following comparison of each sequence to the subtypes and CRFs reference sequences^60^ (database accessed on 8/17/2016), gaps were removed from the final alignments. The phylogenetic tree was constructed by the neighbor-joining and Kimura’s two-parameter methods^61^ using the MEGA.v.5 software^62^. The reliability of the branching orders was determined using 70% bootstrap robustness for subtype assignation^63,64^. Recombination among HIV-1 subtypes was assessed by SCUEAL^65^, COMET^66^, SimPlot^67^, Splitstree^68^, and Rega subtyping tool v.3^69^. The results obtained using each of the five genotyping tools were similar and concordant.

### Determination of drug resistance mutations (DRMs)

DRMs were analyzed in the protease and RT regions using the Stanford algorithm V.8.3^47^ and the International AIDS Society 2015 mutation list was used to confirm each mutation^70^. The prevalence of transmitted DRMs was assessed using the Surveillance Drug resistance Mutations worksheet developed from the 2009 WHO list of mutations for surveillance of transmitted DRMs to NNRTI, NRTI, and PIs^71^. In accordance to these WHO guidelines^71^, the presence of one or more major resistance mutations to any drug class in treatment-naïve patients was considered as transmitted DRM.

### Analysis of Gag P2/NC cleavage site mutations

Mutations were identified in the P2/NC Gag cleavage site of each sequence using HXB2 as reference sequence. Differences in frequency of amino acid sequences and the percentage of patients having each mutation, as compared to the wild type HXB2 virus, were determined following the alignment of all sequences using the CLUSTAL.W integrated into Bioedit.7.2.5 software^58^. Each Gag sequence was analyzed for the presence of specific P2/NC cleavage site mutations known to be associated with resistance to PIs and treatment failure. Samples containing a mixture of wild type and mutant sequences were scored as mutants.

### Statistical analysis

Statistical analysis was performed using GraphPad Prism 5.0b. (GraphPad Software, La Jolla, CA, USA). Data were analyzed by two-tailed unpaired t-test for two groups comparison and P-value ≤ 0.05 was considered statistically significant.

### Data availability

Gag P2/NC and pol nucleotide sequences for all 141 new clinical HIV-1 isolates analyzed in this study are available in the NCBI database; gag GenBank accession numbers: KX894018 – KX894154; pol GenBank accession numbers: KX894155 – KX894279.

### Ethical approval and informed consent

This study was conducted in accordance with the Declaration of Helsinki. The study protocol was approved by the Cameroon National Ethics Committee, as well as the Institutional Review Board of the University of Nebraska Medical Center. All subjects gave written informed consent for inclusion before participating in the study.
